# LMeRAN: Label Masking-Enhanced Residual Attention Network for Multi-Label Chest X-Ray Disease Aided Diagnosis

**DOI:** 10.3390/s25185676

**Published:** 2025-09-11

**Authors:** Hongping Fu, Chao Song, Xiaolong Qu, Dongmei Li, Lei Zhang

**Affiliations:** 1 School of Information Science and Technology, Beijing Forestry University, Beijing 100083, China; fhongping@bjfu.edu.cn (H.F.); songchao1025@bjfu.edu.cn (C.S.); quxiao-long@bjfu.edu.cn (X.Q.); 2 Engineering Research Center for Forestry-Oriented Intelligent Information Processing of National Forestry and Grassland Administration, Beijing 100083, China; 3 National Data Center of Traditional Chinese Medicine, China Academy of Chinese Medical Sciences, Beijing 100083, China; tcmxpzl@126.com

**Keywords:** chest X-ray, computer-aided diagnosis, multi-label classification, attention mechanism, label mask training

## Abstract

Chest X-ray (CXR) imaging is essential for diagnosing thoracic diseases, and computer-aided diagnosis (CAD) systems have made substantial progress in automating the interpretation of CXR images. However, some existing methods often overemphasize local features while neglecting global context, limiting their ability to capture the broader pathological landscape. Moreover, most methods fail to model label correlations, leading to insufficient utilization of prior knowledge. To address these limitations, we propose a novel multi-label CXR image classification framework, termed the **L**abel **M**asking-**e**nhanced **R**esidual **A**ttention **N**etwork (LMeRAN). Specifically, LMeRAN introduces an original label-specific residual attention to capture disease-relevant information effectively. By integrating multi-head self-attention with average pooling, the model dynamically assigns higher weights to critical lesion areas while retaining global contextual features. In addition, LMeRAN employs a label mask training strategy, enabling the model to learn complex label dependencies from partially available label information. Experiments conducted on the large-scale public dataset ChestX-ray14 demonstrate that LMeRAN achieves the highest mean AUC value of 0.825, resulting in an increase of 3.1% to 8.0% over several advanced baselines. To enhance interpretability, we also visualize the lesion regions relied upon by the model for classification, providing clearer insights into the model’s decision-making process.

## 1. Introduction

Thoracic diseases are among the leading causes of morbidity and mortality worldwide. They impose a substantial health burden on individuals and create significant challenges for healthcare systems globally [1]. Early and accurate detection is essential for effective treatment and improved patient outcomes. However, the diagnosis of these conditions often relies heavily on imaging techniques. Among the available modalities, chest X-ray (CXR) plays a pivotal role in detecting thoracic diseases. CXR is widely used because it provides comprehensive information about the lungs and the contents of the chest cavity. It is particularly valuable for the early detection of diseases, enabling timely intervention and management. In radiology research, the interpretation of CXR images has traditionally been performed manually by radiologists. However, this process is both time-consuming and prone to human error, leading to potential misdiagnoses and delayed treatments. To address these challenges, computer-aided diagnosis (CAD) systems have been developed [2,3]. CAD systems utilize advanced models and computational techniques to assist radiologists in the analysis of CXR images, offering rapid and precise analysis, thereby enhancing diagnostic accuracy and efficiency.

**Figure 1:**
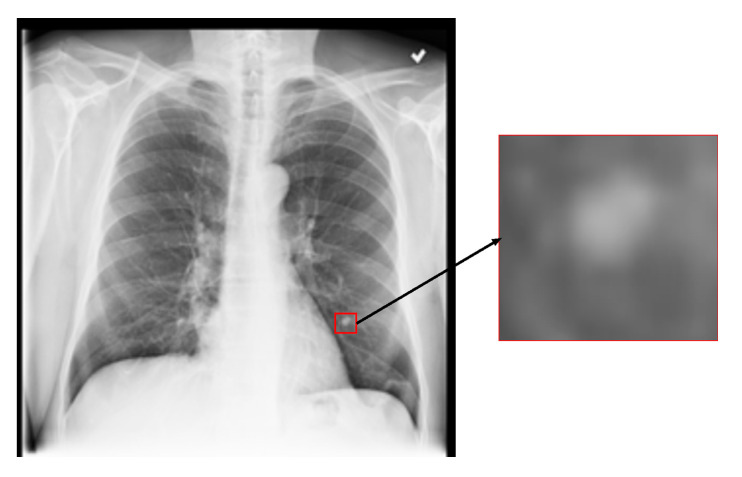
A CXR image with a lung nodule.

Commonly, CXR images are annotated with multiple disease categories, thereby framing the classification task as a multi-label problem. In this context, the predictive model is expected to concentrate on pathological features that are specifically relevant to each label. However, in practical scenarios, non-relevant visual information can interfere with the model’s ability to accurately identify disease-specific patterns. As shown in Figure 1, which presents a CXR image labeled with a “nodule”, the majority of the image comprises normal regions, while the lesion occupies only a small area highlighted by the red box. This limited spatial extent and the subtle nature of the abnormality pose a significant challenge for the model in isolating and learning discriminative features associated with the corresponding disease label.

To tackle the aforementioned issues, numerous recent studies have adopted deep learning-based approaches [4]. For example, Chen et al. [5] combined ResNet [6] and DenseNet [7] in an asymmetric feature learning network to enhance discriminative capacity. Guan et al. [8] proposed ConsultNet, a dual-branch structure that identifies key pathological regions and models disease relationships. Sanida et al. [9] developed a lightweight CNN optimized for embedded systems, achieving high accuracy across seven disease categories. In parallel, Attention mechanisms have also been widely adopted for their ability to focus on relevant features. Saednia et al. [10] used LSTM-based recurrent attention to emphasize abnormal regions. Guan et al. [11] applied localized attention branches to highlight small lesions. Jiang et al. [12] employed self-attention to capture both short- and long-range dependencies. Khater et al. [13] introduced AttCDCNet, integrating attention with DenseNet and focal loss to mitigate class imbalance.

Despite their effectiveness, most attention-based methods tend to overemphasize local lesion features while neglecting global context. This limits their ability to form holistic clinical representations, which are crucial in cases with complex or co-occurring conditions. To address this, we propose the Label Masking-enhanced Residual Attention Network (LMeRAN). Our model incorporates a label-specific residual attention mechanism that combines multi-head self-attention with average pooling, allowing fine-grained lesion features to be highlighted without losing global contextual cues.

In addition, effective multi-label classification must capture dependencies among disease labels. Thoracic diseases often co-occur or influence one another, and modeling these correlations can materially improve predictive accuracy. Masked learning offers a principled way to infer such dependencies from partial observations. In natural language processing, masked language modeling [14] predicts masked tokens from surrounding context, enabling bidirectional context encoding. The idea subsequently migrated to vision as masked image modeling, where random image patches are masked and the network reconstructs tokens or pixels to couple local detail with global structure. Building on this line of work, Lanchantin et al. [15] proposed a label mask training strategy for multi-label image classification to better learn inter-label correlations. We adapt this strategy in LMeRAN, enabling the model to learn latent label dependencies from partially observed labels in a data-driven manner and, in the CXR setting, to exploit disease co-occurrence patterns without requiring explicit prior knowledge.

In this paper, our main contributions can be summarized as follows:

- To the best of our knowledge, this work is the first to incorporate a label mask training strategy into CXR image classification, enabling the model to effectively capture inter-disease correlations and thereby improve both the precision and robustness of predictions.
- We design a novel label-specific residual attention mechanism that simultaneously emphasizes disease-relevant features and retains crucial global image context. Furthermore, we provide visual explanations by highlighting image regions most influential in the model’s decisions, enhancing interpretability and transparency.
- We perform comprehensive evaluations on the publicly available ChestX-ray14 dataset to demonstrate the superiority of LMeRAN and assess the individual contributions of its components through ablation studies.

Section 2 reviews related research in the field. Section 3 introduces the overall design and core components of the proposed LMeRAN model. Section 4 presents the experimental setup along with quantitative evaluations. Section 5 offers an in-depth discussion of the findings. Lastly, Section 6 summarizes the main contributions and concludes the study.

## 2. Related Work

### 2.1. Deep Learning-Based CXR Image Classification

With the continuous improvement of computational resources and the growing availability of large-scale medical datasets, deep learning has become a dominant approach in CXR image classification. A significant milestone was reached in 2017, when Wang et al. [16] introduced the ChestX-ray14 dataset, a large multi-label collection, and assessed the performance of several CNN architectures—AlexNet [17], GoogleNet [18], VGGNet-16 [19], and ResNet—by training only the transition and classification layers. This study set a foundation for subsequent research in automated thoracic disease detection. Building on this, Seibold et al. [20] proposed a method based on ResNet that achieved performance comparable to fully supervised models by training on weakly labeled data. Yao et al. [21] employed a weakly supervised multi-task framework using multi-resolution images to jointly classify and localize thoracic abnormalities, though their method was limited by restricted channel information. To address label noise, Yang et al. [22] introduced a detection strategy that combines features from three networks using an ensemble learning setup. Chen et al. [23] enhanced classification accuracy by modifying the loss function, surpassing the performance of conventional weighted binary cross-entropy. More recently, Ishwerlal et al. [24] proposed an ensemble classification framework with hybrid training optimized by a butterfly algorithm, improving diagnostic accuracy on chest X-rays. Öztürk et al. [25] introduced an adaptive multi-branch transformer that captures disease-specific and shared features for multi-label CXR classification through parallel attention pathways.

### 2.2. Attention in CXR Image Classification

Incorporating attention mechanisms into CXR image classification has proven to be an effective strategy for improving the model’s ability to emphasize diagnostically relevant regions. Ma et al. [26] designed a classification framework based on ResNet-101 that integrated multi-head attention and a squeeze-and-excitation module [27] to enhance inter-channel dependencies, along with a spatial attention module to unify local and global cues. To further refine spatial focus, Guan et al. [28] introduced a residual attention learning model that assigned adaptive weights across different regions of the feature space, thereby enhancing features related to disease and suppressing background noise. Wang et al. [29] proposed a triple attention mechanism within a DenseNet-121 backbone, combining attention modules at the channel, element, and scale levels within a single framework. Taslimi et al. [30] developed SwinCheX, a multi-label classification network utilizing the Swin Transformer [31], which benefited from its hierarchical design and localized self-attention to effectively capture multi-scale visual features in high-resolution CXR images. Peng et al. [32] presented a unified model architecture that integrated local convolution operations with global self-attention, strengthening feature representation. Meanwhile, Wu et al. [33] proposed CTransCNN, which combined CNN and Transformer components to model inter-label relationships via attention-driven feature fusion and multi-branch interaction mechanisms tailored for multi-label medical image classification.

### 2.3. Label Dependency in Multi-Label Image Classification

Effectively capturing label dependencies plays a crucial role in multi-label image classification, especially in the medical domain where multiple diseases often co-exist. Song et al. [34] proposed a multi-modal convolutional network based on multi-instance multi-label learning, utilizing CNN architectures to group labels and exploit contextual associations within those groups. To leverage both sequential and spatial label dependencies, Wang et al. [35] introduced a hybrid CNN-RNN model that jointly learned image-label representations in an end-to-end manner. Lee et al. [36] further explored label correlations using a CNN-GNN hybrid architecture, where graph-based message passing was applied to explicitly model disease co-occurrence patterns in CXR datasets. In parallel, the emergence of CLIP [37] has brought significant advances to multi-label image classification tasks by leveraging its cross-modal alignment capabilities. For example, CLIP-Decoder [38] reduced semantic inconsistency in zero-shot multi-label classification by aligning visual and textual embeddings to better capture inter-label relationships. Additionally, Wang et al. [39] proposed a hierarchical semantic prompt network built upon CLIP, which learned structured label semantics and demonstrated strong performance in settings with sparse positive labels or limited supervision.

## 3. Proposed Method

**Figure 2:**
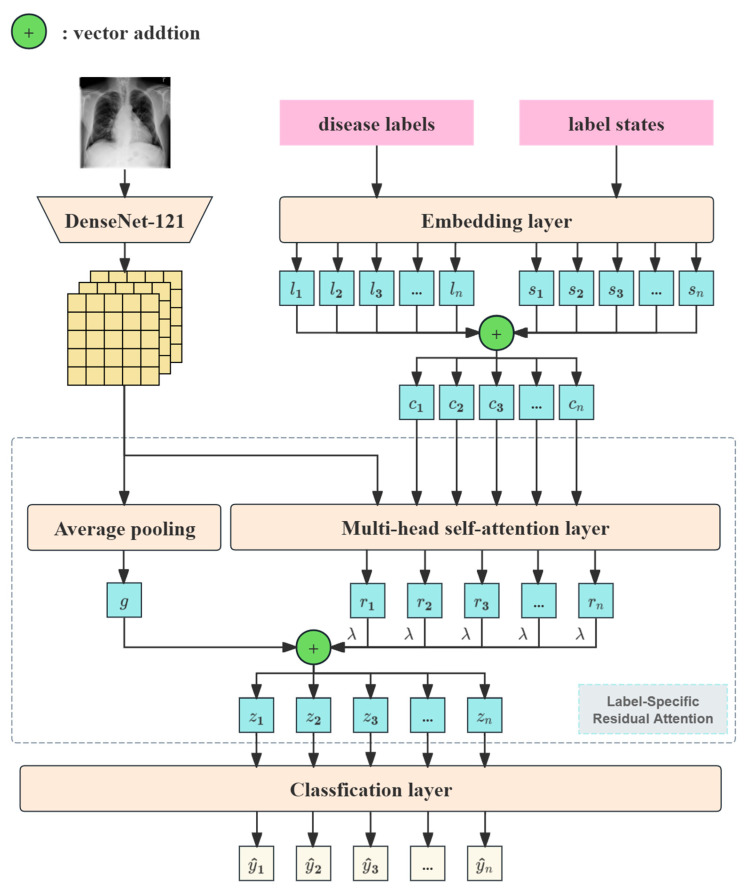
The architecture of LMeRAN.

LMeRAN is a novel multi-label disease classification framework designed to effectively address the challenges associated with diagnostic tasks using chest X-ray images. As illustrated in Figure 2, the framework begins by embedding chest X-ray images, disease labels, and label states into their respective representations. To enrich the label information, label embeddings are combined with their corresponding state embeddings. During training, the state embeddings facilitate the implementation of label masking, enabling the model to capture complex label dependencies. Subsequently, the image feature vectors and the state-enhanced label embeddings are fed into the label-specific residual attention mechanism, which extracts more comprehensive and disease-specific feature representations. Finally, the resulting feature vectors, encoding both global and label-specific information, are passed through a classification layer to predict the probability of disease presence.

### 3.1. Image Feature Extraction

**Figure 3:**
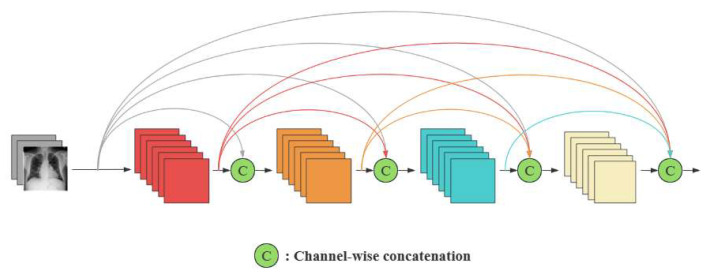
The dense connection mechanism of the DenseNet network.

Robust feature extraction is critical for CXR classification due to the subtle and localized nature of pathological regions. DenseNet [7], characterized by its densely connected architecture, effectively mitigates the vanishing gradient issue and encourages comprehensive feature reuse. In this structure, each layer receives inputs from all preceding layers through feature concatenation, enhancing the propagation of fine-grained information—an advantage in identifying minor lesions or abnormalities across deep layers. As illustrated in Figure 3, such dense connectivity facilitates better information preservation and gradient flow throughout the network. Furthermore, DenseNet’s parameter-efficient design reduces the likelihood of overfitting, a common concern in medical imaging applications with limited annotated data.

In LMeRAN, we employ the DenseNet-121 architecture, pre-trained on the ImageNet dataset, as the backbone for feature extraction. It consists of four dense blocks and three transition layers, offering a compact yet expressive structure. For a given CXR image $X\in R^{H\times W\times D}$, the backbone generates a feature map tensor $F\in R^{h\times w\times d}$, where h, w and d represent the spatial height, width and channel depth, respectively. Each feature vector $f_{i}\in R^{d}$ from F (with i ranges from 1 to h×w) corresponds to a specific subregion in the original image, effectively capturing localized visual cues. These localized representations serve as the input foundation for the attention-based and label-specific analyses performed in subsequent stages of the model.

### 3.2. Embedding Disease Label and State

Beyond embedding CXR images, our model also processes all associated disease labels to generate their respective vector representations, thereby enabling downstream label-specific feature learning. For each input image, we construct a set of label embeddings $L=\{l_{1},\;l_{2},\;l_{3},\cdots ,\;l_{n}\}$, where each vector $l_{i}\in R^{d}$ corresponds to the i-th disease category, and n denotes the total number of disease labels. These label embeddings are learned through a simple embedding layer of size d×l. However, embedding labels alone does not convey the contextual information about the actual state of each label for a given CXR image. To address this limitation, we adopt a straightforward approach by incorporating label states, which provide additional context and knowledge for each label within the CXR image.

Specifically, we generate a set of state embeddings $S=\{s_{1},\;s_{2},\;s_{3},\cdots ,\;s_{n}\}$ through an embedding layer, where each $s_{i}\in R^{d}$ encodes the status of the corresponding disease label. For each label embedding $l_{i}$, we incorporate its state information by summing it with the associated state embedding to form a fused representation:

(1) $$c_{i}=l_{i}+s_{i}$$

where $c_{i}$ denotes the fused embedding that integrates both the semantic identity of the label and its contextual state. The state embedding $s_{i}$ takes on one of three possible states: “unknown”, “negative”, or “positive”. For example, if the label for a given disease is unmasked and confirmed to be present in the image, $s_{i}$ would be correspond to the “positive” state, and its embedding is obtained by inputting the state into the embedding layer.

### 3.3. Label-Specific Residual Attention

To further enhance the image features of disease-related lesion regions and model the relationships between different disease labels, we design a label-specific residual attention mechanism. This mechanism allows the model to independently learn the specific feature representation for each disease, augmenting the disease-related information and the inter-label relationships within the feature representations. Each disease’s feature representation consists of two components: global features and label-specific residual features. The label-specific residual features serve as the residual component to the global features, and both components complement each other to provide a more comprehensive and nuanced representation.

To extract label-specific features, we first process each feature vector $f_{i}\in R^{d}$ from the feature map F. The global feature vector g is calculated by applying average pooling across the entire feature map:

(2) $$g=\frac{1}{h\times w}\sum_{i=1}^{h\times w}f_{i}$$

This global feature vector captures the overall information of the image, providing a contextual summary that complements the label-specific global features. Next, we construct a set $H=\{f_{1},\cdots ,\;f_{h\times w},\;c_{1},\cdots ,\;c_{n}\}$, which includes both the feature vectors from the image and the fused label embeddings. These feature vectors are then passed to a multi-head self-attention layer, where each attention head learns a different subspace of feature relationships. For the t-th attention head, the attention score $\alpha_{ij}^{t}$ between feature vectors $h_{i}\in H$ and $h_{j}\in H$ is first computed. After computing the attention scores for all pairs of feature vectors, we obtain the attention output ${head}_{i}^{t}$ using a weighted summation. Finally, the weighted vectors from multiple attention heads are concatenated along the feature dimension and mapped back to the input dimension through a linear transformation, resulting in the updated feature vector ${\tilde{h}}_{i}$. The overall process can be defined as follows:

(3) $$\alpha_{ij}^{t}=softmax(\left(W_{t}^{q}h_{i}\right){\left(W_{t}^{k}h_{j}\right)}^{T}/\sqrt{d})$$

(4) $${head}_{i}^{t}=\sum_{j=1}^{M}\alpha_{ij}^{t}W_{t}^{v}h_{j}$$

(5) $${\tilde{h}}_{i}=concat({head}_{i}^{1},\;{head}_{i}^{2},\cdots ,\;{head}_{i}^{p})W^{O}$$

where $W_{t}^{q}$, $W_{t}^{k}$, and $W_{t}^{v}$ denote the query, key, and value weight matrices for the t-th attention head, and d is the feature dimensionality. M is the total number of input feature vectors, defined as M=h×w+n. p is the number of attention heads. $W^{o}$ represents the transformation matrix. In addition, this process can be repeated based on the training scenario, where ${\tilde{h}}_{i}$ serves as the input for the next layer. The weight parameters $\left\{W_{t}^{q},\;W_{t}^{k},\;W_{t}^{v},\;W^{o}\right\}$ are not shared across different layers. The final output can be represented as $\tilde{H}=\{{\tilde{f}}_{1},\cdots ,\;{\tilde{f}}_{h\times w},r_{1},\cdots ,\;r_{n}\}$, where $r_{i}$ is the label-specific residual feature vector for the i-th disease label.

To obtain the final feature vector $z_{i}$ for the i-th disease label, we combine the global feature vector g and the label-specific residual feature vector $r_{i}$, weighted by a hyperparameter λ:

(6) $$z_{i}=g+\lambda r_{i}$$

Finally, prediction ${\hat{y}}_{i}$ for the i-th disease label is made using a classification layer, as follows:

(7) $${\hat{y}}_{i}=\sigma (\left(w_{i}\cdot z_{i}\right)+b_{i})$$

where σ is the Sigmoid activation function, $w_{i}$ is the weight vector, and $b_{i}$ is the bias parameter for label i. This output ${\hat{y}}_{i}$ represents the predicted probability for the presence of the i-th disease label.

### 3.4. Label Mask Training Loss

**Figure 4:**
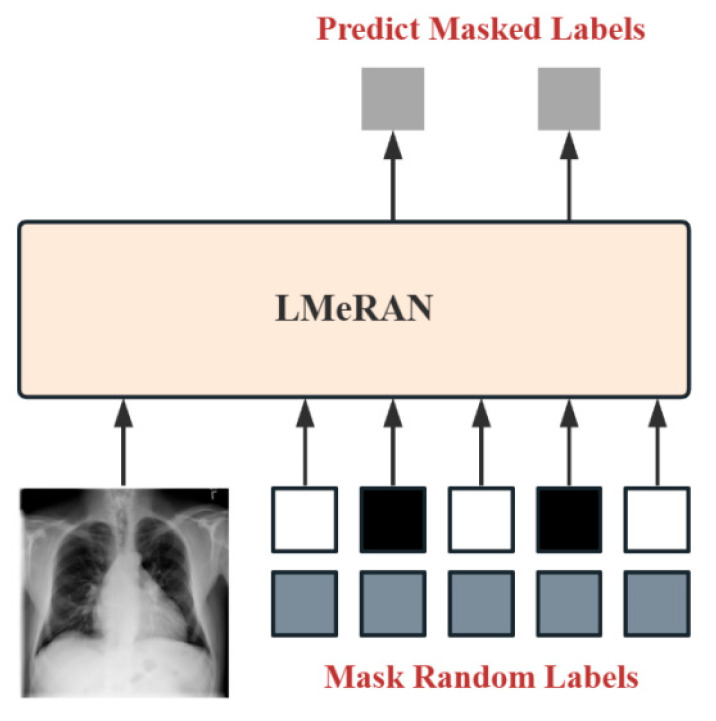
Label mask training process.

State embeddings enable the model to effectively incorporate label knowledge during training. To exploit this capability, we implement a label mask training strategy to help the model learn diverse co-occurrence patterns among labels. As illustrated in Figure 4, a random subset of labels is masked, while the ground truth values of the remaining labels are used to support the prediction of the masked ones.

Suppose there are n possible labels. During training, we randomly select u labels to be masked, where u∈[0.25n,n]. This setting is motivated by two considerations. First, inspired by masked language modeling techniques that typically mask approximately 15% of tokens, we adopt a broader masking range to better accommodate the sparsity and imbalance common in medical multi-label datasets. Second, we aim to expose the model to a wide spectrum of label configurations during training, promoting its ability to learn diverse co-occurrence patterns and reducing its reliance on fixed label subsets.

The masked labels, denoted as $y_{u}$, are randomly sampled from the complete labels y and assigned the embedding corresponding to the “unknown” state. The remaining labels, denoted as $y_{k}$, are treated as known and assigned their respective ground truth state embeddings (“positive” or “negative”). These known labels, along with the input image, are fed into the LMeRAN model, which is trained to predict the masked labels $y_{u}$. Model parameters are optimized using binary cross-entropy loss. By dynamically varying both the number and identity of masked labels during training, the model is encouraged to generalize across a wide range of label combinations, improving robustness in real-world multi-label classification scenarios.

To optimize the model under the label mask training strategy, the loss function is designed to focus solely on the prediction error for masked labels. It is formally defined as follows:

(8) $$L=\sum_{i=1}^{N}E_{p(y_{k})}\{L_{CE}({\hat{y}}_{u}^{\left(i\right)},y_{u}^{\left(i\right)})|y_{k}\}$$

where $L_{CE}$ represents the cross-entropy loss function and $E_{p(y_{k})}\{\cdot |y_{k}\}$ denotes calculating the expectation over the probability distribution of known labels $y_{k}$. N represents the total number of training samples. This loss function ensures that the model does not overfit to specific combinations of known labels, promoting better generalization.

## 4. Experiments

### 4.1. Dataset

We use the large-scale CXR dataset ChestX-ray14 for the experiments. This dataset contains 112,120 frontal view CXR images from 30,805 patients. Among these images, 60,361 are labeled as “No Finding” while the remaining images are labeled as containing one or more thoracic diseases. The distribution of labels in the ChestX-ray14 dataset is shown in Table 1. We observe that the samples of diseases such as hernia, pneumonia, and fibrosis are sparser, while the samples of diseases like infiltration and effusion are more abundant. This imbalanced sample distribution clearly increases the difficulty of model classification.

**Figure 5:**
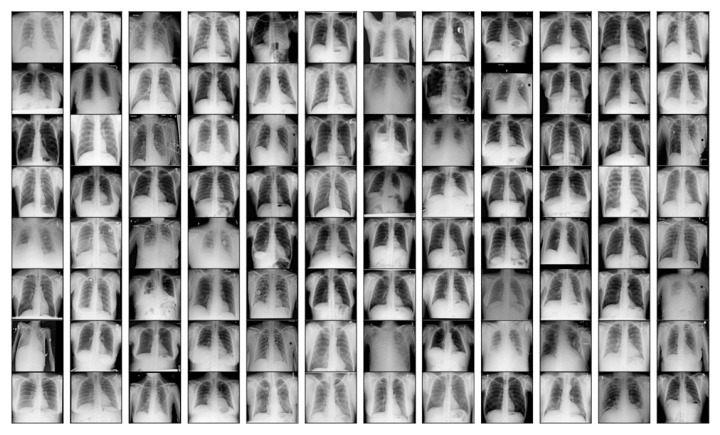
Examples of images in the ChestX-ray14 dataset.

According to the official documentation, we split the dataset, allocating 75,312 images for training, 11,212 for validation, and 25,596 for testing, with a ratio of 7:1:2. Figure 5 shows some examples of CXR images in ChestX-ray14. To speed up the training process, we resize each CXR grayscale image with the original image size of 1024 × 1024 pixels to 448 × 448 pixels by bilinear interpolation. In addition, we employ data enhancement techniques including random cropping, image scaling, and random horizontal flipping to improve the generalization ability of the proposed model.

### 4.2. Evaluation Metrics

We use the receiver operating characteristic (ROC) curve and its area under the curve (AUC) value to evaluate the performance of LMeRAN. The ROC curve’s horizontal axis represents the false positive rate (FPR), while the vertical axis represents the true positive rate (TPR). FPR denotes the proportion of samples with a true label of “0” and incorrectly predicted by the model to be “1”. TPR denotes the proportion of samples with a true label of “1” and correctly predicted by the model to be “1”. The calculation formulas are as follows:

(9) $$FPR\;=\;\frac{FP}{FP\;+\;TN}$$

(10) $$TPR=\frac{TP}{TP+FN}$$

where TP and TN denote the number of correctly predicted positive and negative samples, respectively. FP and FN denote the number of incorrectly predicted positive and negative samples, respectively. Thus, the closer the ROC curve is to the upper left corner, the better the model’s classification performance.

### 4.3. Experiment Setting

The experimental environment consists of a CentOS 7 operating system running on an Intel(R) Xeon(R) Gold 6240 CPU @ 2.60 GHz (Intel Corporation, Santa Clara, CA, USA). Model training is parallelized using two Nvidia RTX2080Ti GPUs (NVIDIA Corporation, Santa Clara, CA, USA), each equipped with 11 GB of VRAM (NVIDIA Corporation, Santa Clara, CA, USA). The LMeRAN model is implemented using the Pytorch framework (version 1.11.0+cu102), with CUDA 10.2 and cuDNN 7.6.5 providing GPU acceleration. Additionally, the model implementation incorporates libraries such as NumPy (version 1.22.3) for numerical computations and SciPy (version 1.9.1) for scientific computing.

The model architecture employs multi-head self-attention layers, with 4 attention heads. A dropout rate of 0.1 is applied to mitigate overfitting, and the parameter λ is set to 0.2 (see Section 4.5.3 for detailed validation). The Adam optimizer, initialized with a learning rate of 0.00001, is used for model training. The training process is conducted over 50 epochs with a batch size of 64. The detailed parameters are summarized in Table 2.

### 4.4. Baselines

To validate the performance of LMeRAN, we compare it with the following five advanced methods:

- Wang et al. [16] use a pre-trained CNN as a feature extractor and focus on training only the transition and classification layers in the model for weakly supervised classification and localization of common thorax diseases.
- Yao et al. [21] employ a multi-resolution analysis approach that combines weakly supervised learning techniques. The model integrates features from different resolutions to enhance the accuracy of medical diagnosis and localization tasks.
- Ma et al. [26] design a multi-attention network for thoracic disease classification and localization, utilizing multiple attention mechanisms to better capture relevant features. The model enhances both classification accuracy and localization precision by focusing on critical areas in the images.
- Peng et al. [32] propose the Conformer model, which merges the local feature extraction capabilities of CNNs with the global representation power of vision transformers. The model effectively captures both local details and long-distance feature dependencies by combining convolution operations with self-attention mechanisms, enhancing representation learning.
- Wu et al. [33] introduce CTransCNN, a model that combines CNNs and Transformers for multi-label medical image classification. It includes a multi-head attention feature module, a multi-branch residual module, and an information interaction module, which together improve label correlation exploration, model optimization, and feature transmission.

### 4.5. Results and Analysis

**Figure 6:**
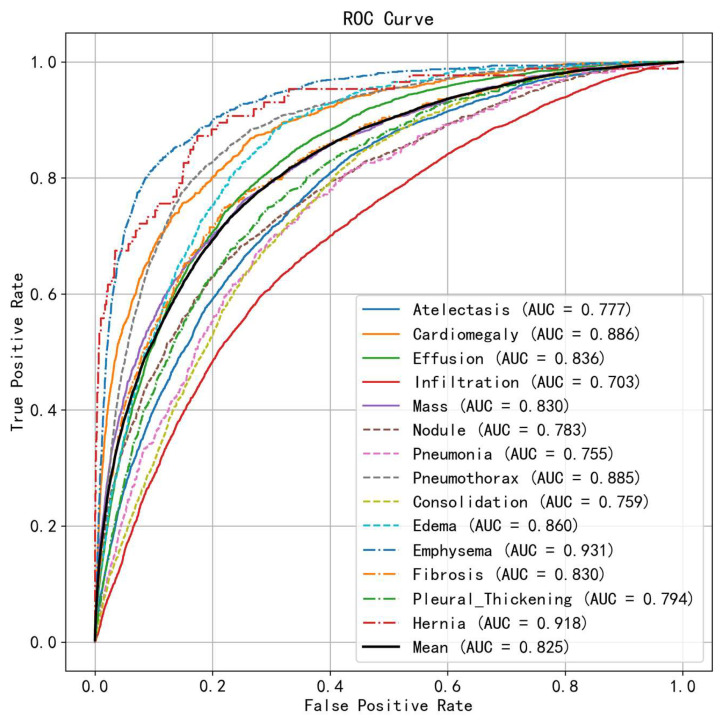
The ROC curves and AUC values for 14 thoracic diseases.

We use ROC curves to visually demonstrate the classification performance of LMeRAN across 14 different thoracic diseases on the ChestX-ray14 dataset, as shown in Figure 6. The figure presents 15 ROC curves: 14 curves for the individual classification performance of LMeRAN for each thoracic disease, and one curve representing the mean classification performance across all diseases. By observing the location of these curves, it can be found that they are concentrated in the upper left corner of the figure, which indicates that LMeRAN shows a high level of accuracy and excellent classification performance in the overall classification of these diseases.

#### 4.5.1. Performance Comparison with Baselines

The AUC values for the classification of various thoracic diseases using different models are presented in Table 3. Our proposed LMeRAN achieves the highest AUC scores for 11 out of 14 thoracic diseases, and for the remaining three conditions—cardiomegaly, effusion, and infiltration—the results are also competitive with the baseline models. Furthermore, in terms of overall classification performance, LMeRAN achieves a mean AUC (mAUC) improvement of 3.1% to 8.0% across 14 thoracic diseases compared to the baseline models, demonstrating its superior classification capability.

#### 4.5.2. Ablation Experiment

To evaluate the impact of the Label Mask Training (LMT) strategy and the Label-Specific Residual Attention (LSRA) mechanism on model performance, we conduct a series of ablation experiments on the ChestX-ray14 dataset. To ensure a fair comparison, all other experimental settings remain consistent across different configurations. The ablation models are defined as follows:

- LMeRAN (w/o LMT): LMeRAN model with the label mask training excluded.
- LMeRAN (w/o LSRA): LMeRAN model with the label-specific residual attention excluded.
- LMeRAN (w/o LMT+ LSRA): LMeRAN model with both the label mask training and label-specific residual attention excluded.
- LMeRAN (Complete): The complete LMeRAN model, including all components.

Table 4 shows the mAUC values obtained by including and excluding the LMT and LSRA components. The following key observations can be drawn from the results:

- LMeRAN (w/o LMT+ LSRA): When both components are excluded, the mAUC is 0.792, serving as the baseline performance.
- LMeRAN (w/o LMT): Including only the LSRA component enhances the mAUC to 0.813. This result highlights that LSRA effectively enhances the model’s ability to focus on discriminative image regions, thereby improving feature representations of disease-relevant areas.
- LMeRAN (w/o LSRA): When only the LMT component is included, the mAUC increases to 0.804. This enhancement suggests that LMT effectively captures the interdependencies among disease labels, allowing the model to leverage label correlations during training to refine its predictions.
- LMeRAN (Complete): When both LMT and LSRA components are included, the mAUC reaches its highest value of 0.825. This confirms that the joint contribution of both components leads to superior classification performance, effectively combining label dependency modeling and enhanced image feature representation to maximize predictive accuracy.

**Table 4:** Ablation experiment results. A “√” indicates the inclusion of the component, while a “×” denotes its exclusion.

	LMT	LSRA	mAUC
LMeRAN (w/o LMT+ LSRA)	×	×	0.792
LMeRAN (w/o LMT)	×	√	0.813
LMeRAN (w/o LSRA)	√	×	0.804
LMeRAN (Complete)	√	√	0.825

#### 4.5.3. Parameter Sensitivity Experiment

To further evaluate the robustness of LMeRAN, we conduct parameter sensitivity experiments focusing on three key factors: the balance parameter λ in LSRA, the number of attention heads (H), and the lower bound of the mask ratio (R) in LMT. Unless otherwise specified, all other experimental settings, including backbone, optimizer, training schedule, and data split, are kept consistent with the main experiments.

**Figure 7:**
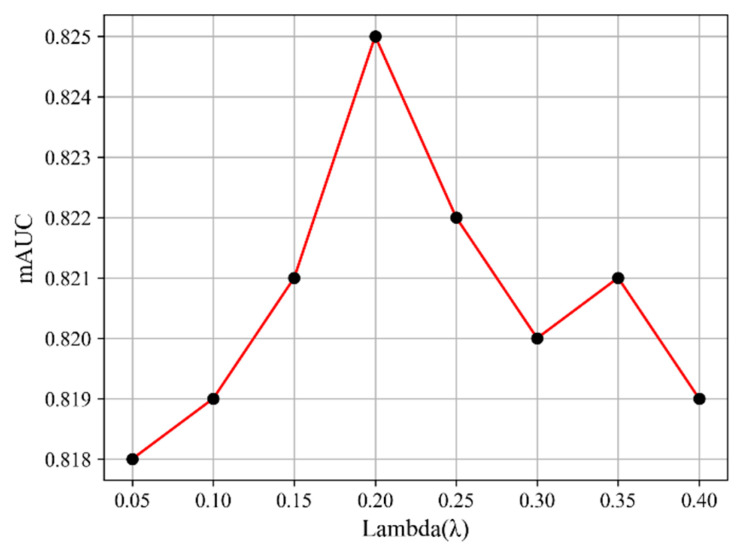
Sensitivity of LMeRAN to the balance parameter λ on ChestX-ray14.

The effect of λ is shown in Figure 7. As λ increases from 0.05 to 0.20, the mAUC of LMeRAN rises steadily and reaches a peak of 0.825 at λ = 0.20. Beyond this point, performance begins to decline, indicating that excessive reliance on self-attention while weakening pooling degrades classification accuracy. The results also show that LMeRAN remains relatively stable when λ lies within [0.15, 0.25], but becomes sensitive at extreme values.

The impact of varying the number of attention heads is presented in Table 5. With a single head, the model achieves an mAUC of 0.819. Increasing to 2 and 4 heads improves performance to 0.822 and 0.825, respectively. When the number of heads is further increased to 6 or 8, the mAUC remains stable at 0.824, showing no additional benefit. These results suggest that too few heads limit feature diversity, while excessive heads introduce redundancy without improving accuracy. A moderate number of heads (H = 4 or H = 6) provides a favorable balance between representational capacity and efficiency.

The effect of the mask ratio lower bound is summarized in Table 6. The model achieves the best performance (mAUC = 0.825) when R = 0.25. Reducing the lower bound to R = 0.00 decreases performance to 0.818, as overly weak masking exposes too many ground-truth labels and restricts the learning of label co-occurrence patterns. Conversely, increasing R to 0.50 and 0.75 reduces mAUC to 0.821 and 0.817, respectively, because excessive masking diminishes supervision and destabilizes optimization. These results highlight that moderate masking strength encourages effective co-occurrence modeling while preserving sufficient supervision.

Overall, these parameter sensitivity experiments demonstrate that LMeRAN maintains stable performance across a reasonable range of λ, H, and R, while moderate settings (λ = 0.20, H = 4, R = 0.25) provide the most effective trade-off between accuracy, robustness, and efficiency. However, it should be noted that these values are derived from experiments on ChestX-ray14 and may not directly generalize to other datasets or tasks. Further validation on diverse benchmarks is necessary to confirm their applicability, and ongoing refinements to the model may help identify parameter configurations that are more broadly effective.

#### 4.5.4. Interpretability Analysis

**Figure 8:**
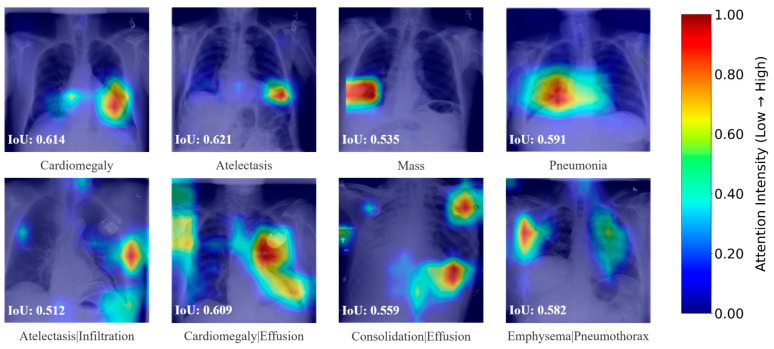
Lesion localization heatmaps.

In addition to evaluating classification accuracy, we conducted an interpretability analysis to gain further insights into the model’s diagnostic behavior. Specifically, we utilize attention weight matrices to generate lesion localization heatmaps, which highlight the regions of CXR images deemed most relevant by the model during prediction. As illustrated in Figure 8, these heatmaps visually identify the areas contributing most significantly to the classification decisions for various thoracic conditions. To verify the clinical relevance of the highlighted regions, we not only invited medical experts to visually evaluate the attention maps, but also introduced a quantitative assessment using the Intersection over Union (IoU). Specifically, we defined the pixels with attention intensity greater than 0.4 as pseudo-segmentation regions and calculated their IoU with the ground-truth lesion annotations. This dual evaluation strategy further confirms the validity and reliability of the extracted features. Furthermore, the visual interpretability afforded by these heatmaps not only aids clinicians in understanding the model’s predictions but also enhances the transparency of the CAD system, thereby fostering trust and supporting its integration into medical practice.

## 5. Discussion

Experimental results demonstrate that LMeRAN achieves superior classification performance on CXR images compared with several representative methods. We benchmark our model against five baselines: Wang et al. [16], Yao et al. [21], Ma et al. [26], Conformer [32], and CTransCNN [33]. Compared with Wang et al. and Yao et al., LMeRAN improves the mAUC by 8.0% and 6.4%, respectively, highlighting the contribution of attention-enhanced representations to classification robustness. Relative to Ma et al. and CTransCNN, which primarily emphasize local lesion features, LMeRAN yields additional mAUC gains of 3.1% and 4.0% by jointly integrating global context and local discriminative features. The advantage is particularly evident for diseases with diffuse or context-dependent manifestations, such as Mass (+2.9% and +4.9%) and Pleural Thickening (+4.0% and +10.4%). These findings indicate that balancing global and local information enables more accurate and reliable predictions than models biased toward localized lesion representations. Moreover, when compared with Conformer, LMeRAN improves the mAUC by 6.1%, further validating the effectiveness of the proposed LSRA mechanism. In addition, the integration of the LMT strategy allows the model to better capture inter-label dependencies.

While these results confirm the effectiveness of LMeRAN, they are currently based solely on the ChestX-ray14 dataset. Although this benchmark remains widely used, it has certain limitations in terms of scale and diversity. We will further evaluate LMeRAN on larger and more diverse datasets such as CheXpert and MIMIC-CXR to provide stronger evidence of its generalizability and robustness across different clinical scenarios.

## 6. Conclusions

In this study, we propose a novel multi-label medical image classification model, called LMeRAN, specifically designed for the CXR image classification task. LMeRAN introduces two primary innovations: (1) a label-specific residual attention mechanism that integrates an average pooling layer with a multi-head self-attention layer to extract comprehensive feature representations for multiple diseases in CXR images, and (2) a label mask training strategy that enables the model to explore a wide range of label co-occurrences. Extensive experiments conducted on the large-scale, publicly available ChestX-ray14 dataset demonstrate that LMeRAN offers a significant performance advantage over existing approaches. Furthermore, qualitative assessments using lesion localization heatmaps underscore the model’s reliability and interpretability.

Future work will follow three main directions. First, we will further address the challenge of label imbalance. In addition to conventional augmentation, we plan to investigate cost-sensitive loss, reweighting strategies, and generative augmentation methods to improve the performance of minority classes. Second, to strengthen clinical applicability and ensure generalization, we will extend validation to additional external datasets and real-world hospital data. Third, we aim to integrate multimodal information, such as clinical notes or patient metadata. This extension may provide richer contextual signals and further enhance diagnostic accuracy. Collectively, these efforts will guide the development of LMeRAN into a robust and clinically adaptable framework for multi-label chest X-ray analysis.

## Figures and Tables

**Table 1:** Label distribution in the ChestX-ray14 dataset.

Labels	Quantity	Frequency
No Finding	60,361	42.65%
Infiltration	19,894	14.06%
Effusion	13,317	9.41%
Atelectasis	11,559	8.17%
Nodule	6331	4.47%
Mass	5782	4.09%
Pneumothorax	5302	3.75%
Consolidation	4667	3.30%
Pleural Thickening	3385	2.39%
Cardiomegaly	2776	1.96%
Emphysema	2516	1.78%
Edema	2303	1.63%
Fibrosis	1686	1.19%
Pneumonia	1431	1.01%
Hernia	227	0.16%

**Table 2:** Parameter setting.

Parameters	Value
optimizer	Adam
learning_rate	0.00001
dropout_rate	0.1
batch_size	64
epoch	50
number of layers	4
number of heads	4
λ	0.2

**Table 3:** Experimental results of different models. **Bold numbers** indicate the best results, while *underlined italics* represent the second-best results.

Disease	Wang et al. [16]	Yao et al. [21]	Ma et al. [26]	Conformer [32]	CTransCNN [33]	LMeRAN (Ours)
Atelectasis	0.700	0.733	*0.763*	0.727	0.748	**0.777**
Cardiomegaly	0.810	0.865	0.884	**0.907**	*0.900*	0.886
Effusion	0.759	0.806	0.816	0.806	**0.837**	*0.836*
Infiltration	0.661	0.673	0.679	0.697	**0.707**	*0.703*
Mass	0.693	0.718	*0.801*	0.761	0.781	**0.830**
Nodule	0.669	*0.777*	0.729	0.728	0.742	**0.783**
Pneumonia	0.658	0.684	*0.710*	0.623	0.630	**0.755**
Pneumothorax	0.799	0.805	0.838	0.831	*0.847*	**0.885**
Consolidation	0.703	0.711	*0.744*	0.700	0.731	**0.759**
Edema	0.805	0.806	0.841	0.828	*0.858*	**0.860**
Emphysema	0.833	0.842	*0.884*	0.815	0.856	**0.931**
Fibrosis	0.786	0.743	0.801	*0.804*	0.778	**0.830**
Pleural Thickening	0.684	0.724	*0.754*	0.681	0.690	**0.794**
Hernia	0.872	0.775	0.876	0.786	*0.881*	**0.918**
mAUC	0.745	0.761	*0.794*	0.764	0.785	**0.825**

Note: Improvements over the baselines are evaluated using the DeLong test for AUC. All reported gains of LMeRAN over comparison methods are statistically significant (*p* < 0.05).

**Table 5:** Sensitivity of LMeRAN to the number of attention heads on ChestX-ray14.

	H = 1	H = 2	H = 4	H = 6	H = 8
mAUC	0.819	0.822	0.825	0.824	0.824

**Table 6:** Sensitivity of LMeRAN to the mask ratio lower bound on ChestX-ray14.

	R = 0	R = 0.25	R = 0.50	R = 0.75
mAUC	0.818	0.825	0.821	0.817

## Data Availability

The dataset used in this study is publicly available and can be accessed at: http://nihcc.app.box.com/v/ChestXray-NIHCC (accessed on 15 June 2025).
